# New Vectors of TTX Analogues in the North Atlantic Coast: The Edible Crabs *Afruca tangeri* and *Carcinus maenas*

**DOI:** 10.3390/md21060320

**Published:** 2023-05-25

**Authors:** Sandra Lage, Felicitas ten Brink, Adelino V. M. Canário, José P. Da Silva

**Affiliations:** 1 Centre of Marine Sciences (CCMAR/CIMAR LA), University of Algarve, Campus de Gambelas, 8005-139 Faro, Portugal; 2 Energy and Environment Institute, School of Environmental Sciences, University of Hull, Hull HU6 7RX, UK

**Keywords:** emergent toxins, seafood safety, occurrence data, European waters, LC–HRMS

## Abstract

Tetrodotoxin (TTX) and its analogues are naturally occurring toxins historically responsible for human poisoning fatalities in Eastern Asia. It is typically linked to the consumption of pufferfish and, to a lesser extent, marine gastropods and crabs. In the scope of a comprehensive project to understand the prevalence of emergent toxins in edible marine organisms, we report, for the first time, the detection of TTX analogues in the soft tissues of edible crabs, the European fiddler crab *(Afruca tangeri*) and green crab (*Carcinus maenas*), harvested in southern Portugal. No TTX was detected in the analyzed samples. However, three TTX analogues were detected—an unknown TTX epimer, deoxyTTX, and trideoxyTTX. These three analogues were found in the European fiddler crab while only trideoxyTTX was found in the green crab, suggesting that the accumulation of TTX analogues might be influenced by the crabs’ different feeding ecology. These results highlight the need to widely monitor TTX and its analogues in edible marine species in order to provide adequate information to the European Food Safety Authority and to protect consumers.

## 1. Introduction

Tetrodotoxin (TTX) is a low-molecular-weight, water-soluble neurotoxin with approximately 30 known structural analogues, collectively known as tetrodotoxins (TTXs), named after the Tetraodontidae puffer fish family, from which they were initially isolated (Table 1) [1]. It is known that TTX toxicity depends on the molecular structure and its affinity to sodium channels of excitable tissues [1,2]. However, the information on the relative potencies of individual TTX analogues is still limited [3]. Intoxication by TTX is common in Japan, Taiwan, Bangladesh, and Southeast Asia, and is caused by eating puffer fish and other marine organisms such as gastropods and crabs [2,4,5,6,7,8,9,10] which could be TTX vectors and pose a food safety risk in the European Union (EU). Other species belonging to different phyla of marine and terrestrial animals have also been shown to contain TTXs [2,8]. The widespread distribution of TTXs has been related to exogenous sources, specifically accumulation via symbiotic hosting of a TTX producer and food chain transmission [11,12]. The origin of TTXs is thought to be a result of bacterial metabolism, namely from the genera *Pseudomonas*, *Pseudoalteromonas*, and *Vibrio*, although it has also been reported in Actinobacteria, Bacteroides, Firmicutes, and Proteobacteria [13,14].

TTX bearers are typically found in tropical ecosystems of Eastern Asia. However, in recent years, TTX started to be detected in Mediterranean and Atlantic puffer fish, bivalve molluscs, marine gastropods, and echinoderms [7,15,16,17,18,19]. To ensure consumer protection in the EU, the market placement of fish species described as TTX bearers (*Tetraodontidae*, *Canthigasteridae*, *Molidae*, and *Diodontidae*) is prohibited by European legislation [20,21]. However, the first and only known TTX human intoxication event reported in the EU was not due to the ingestion of fish, but of a marine gastropod, the trumpet shell *Charonia lampas* (Linnaeus, 1758) (containing 315,000 µg/kg TTX in the digestive gland) presumably caught off the Algarve coast, south of Portugal [7]. Since then, TTX was also detected in bivalve molluscs from Greece, the UK, and the Netherlands [17,19,22], which has led the European Food Safety Authority (EFSA) to recommend a maximum safe limit of 44 µg TTX equivalent (eq)/kg of shellfish meat [3]. More recently, TTX concentrations 7- to 12-fold higher than this limit were reported in bivalve molluscs from Italy [23,24]. However, TTX and its analogues are still not regulated or regularly monitored in the EU.

Recent studies of TTX seasonal variability in Portugal and Spain concluded that TTX accumulation by bivalves does not pose a risk to consumers, since only trace levels (below limit of quantification, LOQ) were reported in most of the samples analyzed [15,25,26,27]. These results contrast with the high TTX values (above the EFSA-recommended limit) determined in benthic marine gastropods (up to 315,000 µg/kg) and echinoderms (up to 352,886 µg/kg) along the Portuguese continental platform and the Azores archipelago [7,15,16,28,29]. Nevertheless, as stated in the EFSA’s scientific opinion, more data are still required to make a reliable TTX exposure assessment and effectively protect consumer health in the EU [3].

The present study was developed in the scope of an emergent toxins monitoring project regarding edible marine organisms of the Portuguese coast with the purpose to build reliable knowledge to support decision making and consumer health. Here, we identify TTX analogues in the edible European fiddler crab *Afruca tangeri* (Eydoux, 1835) and green crab *Carcinus maenas* (Linnaeus 1758) from southern Portugal, and report for the first time TTX analogues at concentrations above the EFSA-recommended limit in crabs harvested in the EU.

## 2. Results

**Figure 1:**
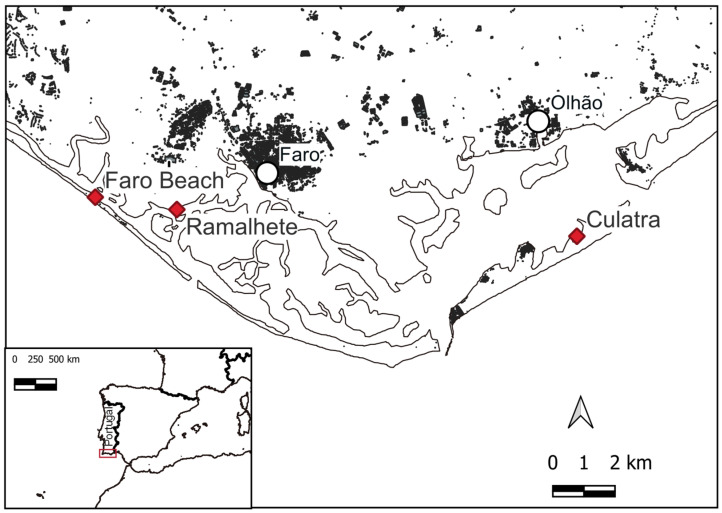
Location of the sampling points in the Ria Formosa lagoon, southern Portugal: Ramalhete Marine Station, Faro beach, and Culatra Island indicated by the red rhombuses.

**Figure 2:**
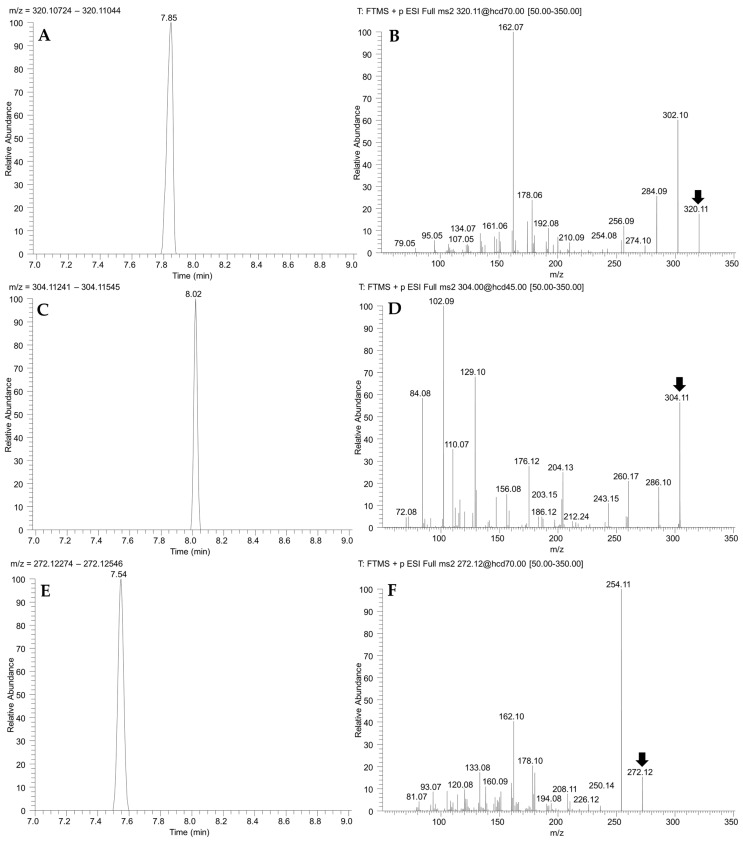
Accurate mass-extracted ion chromatograms (AM-XIC) and mass spectra of TTX analogues detected in the soft tissues of European fiddler crab harvest near Faro beach. AM-XIC were generated from the LC–HR-MS full-scan positive mode (ESI+) chromatograms using the theoretical TTX analogues *m*/*z* with a ±5 ppm extraction window. Mass spectra were obtained under a product ion scan with higher-energy collisional dissociation (HCD) fragmentation of the TTX analogues, over the entire chromatographic separation (HCD MS^2^) and using a normalized collision energy (CE). Fragment detection had a *m/z* window of 50 to 350. (**A**) AM-XIC taken at *m*/*z* 320.10884 (TTX epimer). (**B**) HCD MS^2^ spectrum of TTX epimer, 70 CE. (**C**) AM-XIC taken at *m*/*z* 304.11393 (deoxyTTX). (**D**) HCD MS^2^ spectrum of deoxyTTX, 45 CE. (**E**) AM-XIC taken at *m*/*z* 272.12410 (trideoxyTTX). (**F**) HCD MS^2^ spectrum of trideoxyTTX, 70 CE. Arrow indicates the TTX analogue theoretical *m*/*z* being fragmented.

A total of 24 individuals, collected at three sampling points (Ramalhete Marine Station, Faro beach, and Culatra Island) in the Ria Formosa lagoon, southern Portugal (Figure 1), were pooled into 12 samples (each sample containing the soft tissues of 2 individuals of the same species and sampling point). Samples were screened for the presence of TTX and its analogues (Table 1) using liquid chromatography–high-resolution mass spectrometry (LC–HRMS) [2,30]. The identification of TTX and its analogues was based on the exact masses of their protonated molecular ions [M+H]^+^ (*m/z*, see Table 1), retention times and on their fragmentation spectra obtained by higher-energy collisional dissociation (HCD). Due to the unavailability of certified reference materials for TTX analogues, the concentration of the detected compounds was estimated using the instrumental response to TTX (Figure 2).

The limits of detection and quantification (LOD and LOQ) for TTX in the crabs’ soft tissue matrix were 5.0 and 16.6 µg/kg, respectively. A strong ion suppression was noted for TTX in the crabs’ soft tissues matrix; the matrix effect (ME) was 52.6 ± 5.38%.

**Figure 3:**
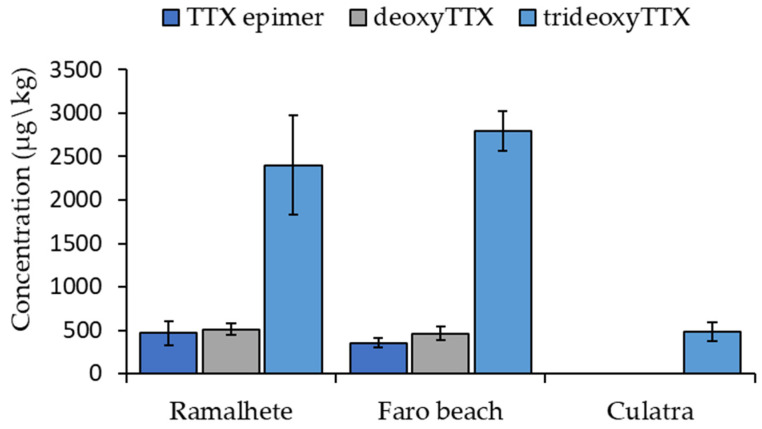
Concentration (mean ± standard deviation, *n* = 4) of the TTX analogues: TTX epimer, deoxyTTX, and trideoxyTTX (µg/kg) in the European fiddler crab (from Ramalhete Marine Station and Faro beach) and green crab (from Culatra Island) soft tissues.

No TTX was detected in the analyzed tissues. However, three analogues, an unknown TTX epimer, deoxyTTX and trideoxyTTX, were detected in all European fiddler crab soft tissue samples collected near Faro beach (Figure 2 and Figure 3). The AM-XIC obtained with the *m*/*z* 320.10884 (theoretical exact mass of TTX and epimers protonated molecular ions) of these samples had a peak with a retention time, which did not correspond to the retention times of TTX or its epimer 4-*epi*TTX (Supplementary materials Figure S1). However, the fragmentation spectra produced by HCD contained diagnostic TTX fragments (Figure 2B). As a result, this compound was identified as a hitherto unknown TTX epimer. The concentrations of the unknown TTX epimer, deoxyTTX and trideoxyTTX in the European fiddler crab soft tissue samples collected near Faro beach ranged from 254.8 to 537.3 µg/kg, 419.8 to 725.0 µg/kg, and 2245.0 to 3458.5 µg/kg, respectively. Two of the four pooled samples of European fiddler crab collected at Ramalhete Marine Station contained these three analogues above the LOQs (Figure 3). The other two samples had only deoxyTTX and trideoxyTTX above the LOQs; the unknown TTX epimer was present but below the LOQ. At this sampling point, the concentrations of the unknown TTX epimer, deoxyTTX and trideoxyTTX were similar to the concentrations found at Faro beach; and ranged from 268.9 to 663.6 µg/kg, 295.8 to 667.3 µg/kg and 1344.9 to 4232.5 µg/kg, respectively. In the green crab samples, only trideoxyTTX was detected in animals caught at Culatra Island, and its concentrations were above the LOQs in just two samples (i.e., 334.6 and 629.7 µg/kg). These concentrations were 5-fold lower than in European fiddler crab collected near Faro beach and at Ramalhete Marine Station (Figure 3). In the European fiddler crab, trideoxyTTX concentrations were 5-fold higher than the concentrations of TTX epimer and deoxyTTX (Figure 3).

**Figure 4:**
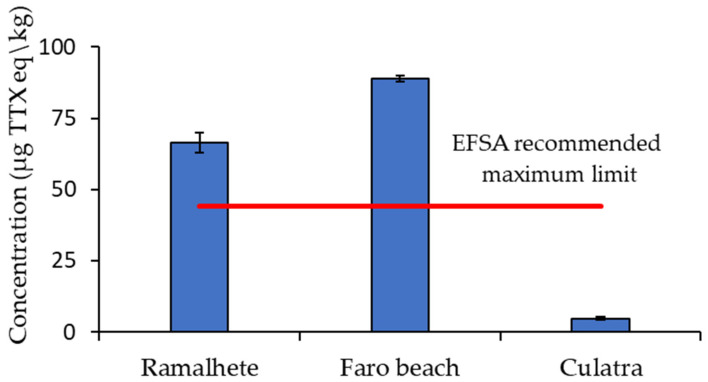
Total concentration (mean ± standard deviation, *n* = 4) in µg TTX equivalent (eq)/kg in the European fiddler crab (from Ramalhete Marine Station and Faro beach) and green crab (from Culatra Island) soft tissues.

The total TTX concentration (µg TTX eq/kg) of the European fiddler crab soft tissues at Ramalhete Marine Station and Faro beach was 1.5- and 2-fold higher than the EFSA-recommended safety limit of 44 µg TTX eq/kg, respectively (Figure 4). However, in the green crab it was 16-fold lower than in European fiddler crab, and below the recommended safety limit (Figure 4). The relative potencies of 0.16 and 0.01 were used for the unknown TTX epimer and deoxyTTX/trideoxyTTX, respectively, to determine the total TTX concentration, as recommended by the EFSA for 4-*epi*TTX and 5,6,11-deoxyTTX/5,6,11- trideoxy TTX [3,31,32,33].

## 3. Discussion

To the best of our knowledge, this is the first report identifying TTX analogues in the edible European fiddler crab and green crab. These findings are significant as they provide new occurrence data of TTXs in marine organisms, and they contribute to a better assessment of the risk these emergent marine toxins pose to human health. Moreover, the presence of TTXs above the EFSA-recommended safety limit of 44 µg TTX eq/kg highlights a continued risk to food consumers from TTXs in the Algarve region [3]. European fiddler crabs and green crabs are caught by shellfish gatherers both for direct consumption and for sale at markets and restaurants, as well as for use as live bait for fishing. While the European fiddler crab’s large male claws are a delicacy mostly caught and consumed locally, the green crab is of high economic importance in the EU and is harvested along the entire Portuguese coast [34].

In the European fiddler crab samples from both Faro beach and Ramalhete Marine station, three TTX analogues (unknown TTX epimer, deoxyTTX, and trideoxyTTX), but no TTX, were found. In contrast, in green crabs obtained from Culatra Island, trideoxyTTX was the only analogue detected, and at 5-fold lower concentrations than in European fiddler crabs. This resulted in samples exceeding the EFSA-recommended safety limits of TTX concentration in the European fiddler crab but not in the green crab. Given that the two leading hypotheses for TTX accumulation in organisms are symbiotic hosting of a TTX producer and food chain transmission [11,12,35,36], it is possible that the observed differences in TTX concentration between species are due to their feeding ecology. The European fiddler crab feeds only during low tide (both at night and day), and mostly on sediment (which contains bacteria, microphytobenthos and detritus), as well as macroalgae, salt marsh plants and animal carcasses [37,38]. The green crab is a voracious, opportunistic predator that feeds on a wide variety of prey and is mostly active at night and at high tide. Its diet can change dramatically due to seasonal changes in diversity and abundance of prey species as well as preys body size and age, but it is primarily composed of molluscs, crustaceans, and polychaetes. However, it is important to note that juvenile green crabs, such as those used in this study, may also consume biofilm from the sediment surface [39,40,41], but to a lesser extent. Therefore, the higher TTX concentrations in European fiddler crab may be due to TTX-producing bacteria either inhabiting their intestines or the marine sediments the crabs feed on. Evidence for TTX producing intestinal bacteria has been found in TTX-bearing crabs from Eastern Asia [42,43,44], i.e., the xanthid crabs *(Xanthidae*, primarily *Zosimus aeneus* (Linnaeus, 1758), *Atergatis floridus* (Linnaeus, 1767) and *Platypodia granulosa* (Rüppell, 1830)), which are nocturnal and omnivorous crabs feeding on sediment, macroalgae, sponges, corals, bivalve molluscs, and gastropods [2,6,9,45]. Moreover, actinobacteria isolated from marine sediments from Tokyo Bay and the Pacific Ocean have been shown to produce TTXs [46], suggesting that TTX-bearing crabs may get exposed to TTX from the sediment. Furthermore, recent studies on the TTX-bearer gastropod, grey side-gilled sea slug *Pleurobranchaea maculata* (Quoy and Gaimard, 1832), from New Zealand have provided evidence on a dietary origin of TTX and suggested that a symbiotic microbial source of TTX is unlikely in these organisms [35,36]. Future research should focus on the potential TTX production by bacteria in both the European fiddler crab intestines and the sediment.

TrideoxyTTX concentration in the European fiddler crab samples was 5-fold higher than the concentrations of the other analogues, and trideoxyTTX was the only analogue detected in green crab. Thus, contrary to previously assumptions, TTX analogues can be detected in the absence of detectable levels of the parent toxin [47]. Similarly, two trideoxyTTX isomers, but no TTX above LOQ, were detected in the crab *Liocarcinus corrugatus* (Pennant, 1777) harvested at the Galician Atlantic coast (northwest Spain) [48]. This is consistent with our previous findings in the trumpet shell *Charonia lampas* (a gastropod) caught off the Algarve coast, in which trideoxyTTX accounted for approximately 19% of the TTX profile in several tissues while TTX accounted for only 1–2% [29]. An up to 3-fold higher concentration of 5,6,11-trideoxyTTX in relation to TTX was also reported for trumpet shell digestive gland caught at a nearby location [7]. Furthermore, the entire trumpet shell soft body harvested at the northwest Portuguese coast exhibited a 5,6,11-trideoxyTTX concentration of 6 µg/kg, but no TTX was detected [15]. This same pattern of detection of trideoxyTTX with no or lower concentration of TTX was also reported in another study of marine gastropods (off Portuguese coast) and puffer fish (from the Azores archipelago) [16]. Thus, trideoxyTTX is a major analogue of TTX in puffer fish, gastropods and crabs caught in Portuguese waters, just as previously suggested for puffer fish and marine gastropods from Eastern Asia [49,50,51,52]. Interestingly, 5,6,11-trideoxyTTX is hypothesized to be a precursor of TTX in TTX-producing marine bacteria [51]. Although the biosynthetic and metabolic pathways of TTX are still unknown, they are thought to involve a series of oxidations, i.e., 5,6,11-trideoxyTTX → 5,11-dideoxyTTX or 6,11-dideoxyTTX → 5-deoxyTTX, 11-deoxyTTX or 6-deoxyTTX → TTX → 11-norTTX-6-ol → 11-oxoTTX [53,54,55]. The possibility of trideoxyTTX being oxidized to TTX within the marine organisms remains to be elucidated. Furthermore, the accumulation mechanism or exact metabolic pathway of TTXs in crabs remains unclear.

The LOD, LOQ, and ME for TTX in the pooled soft tissue samples used in this study, are within the values previously reported by us for individual trumpet shell tissues (a gastropod) using the same extraction and analysis method [29]. In the gastropod, the LOD and LOQ ranged from 1.2 to 43.7 µg/kg and 4.1 to 151.47 µg/kg, respectively. ME ranged from 88.6 ± 4.69% (ion suppression) in the mantle to 380.5 ± 12.18% (ion enhancement) in the stomach [29]. Thus, ME is highly tissue dependent, and its determination is critical in LC–MS quantitative studies. The ME detected in our soft tissue samples reflect the pooled average of all crab tissues and are thus not representing the full spectrum of matrix variations that may occur across tissues. Furthermore, because of their structural similarity, TTX analogues are assumed to have an equivalent ME to TTX. However, because almost all TTX analogues are not commercially available, it remains to be confirmed. Another downside of pooling samples is the dilution effect, which occurs when samples with quantifiable analyte concentrations are mixed with samples with no or low analyte concentrations, resulting in a pooled sample with non-quantifiable concentration. This could have happened in our pooled samples, where the TTX analogues were detected but their concentration was below LOQ.

Our results highlight the importance of routine preventative monitoring of marine toxins and their analogues in different commercial species to identify new vectors and providing adequate health protection within the EU.

## 4. Materials and Methods

### 4.1. Sampling

European fiddler crab, *Afruca tangeri* (Eydoux, 1835), and green crab, *Carcinus maenas* (Linnaeus 1758), specimens were captured along the intertidal zone of Ria Formosa lagoon (Figure 1) at the end of June 2021 (week 26) during low or receding tides and at mid to low coast height. Eight European fiddler crab individuals were collected in the vicinity of Faro beach (37°00′35.9″ N 7°59′36.3″ W) and another eight at Ramalhete Marine station (37°00′23.5″ N 7°58′10.5″ W). Eight green crab specimens were collected from Culatra Island (36°59′40.7″ N 7°49′51.0″ W) (Figure 1). Within two hours of sampling, the samples were brought to the CCMAR facilities.

The carapace length (CW) of the specimens was measured using precision vernier calliper to the nearest 0.01 mm, and the wet weight (WW) was determined using a digital scale to the nearest 0.01 g. The CW and WW of European fiddler crab collected at Faro beach were 26.6 ± 2.41 mm and 14.4 ± 1.95 g, respectively, and 24.3 ± 5.19 mm and 11.7 ± 3.97 g for specimens collected at Ramalhete Marine Station. The CW and WW of green crab were 29.8 ± 4.63 mm and 12.5 ± 2.71 g, respectively. The crabs were dissected and gills, midgut gland, gonads, stomach, heart, and hypodermis under the carapace were pooled together with muscle from the claws, legs, and general body. The soft tissues of two individuals of the same species from the same sampling site were combined to make a pooled sample (4 pooled samples per station). This pooling was done to obtain the required tissue weight to perform the TTX extraction according to the Standard Operating Procedure of the European Union Reference Laboratory for Marine Biotoxins [56]. The samples were kept at −20°C until they were processed.

### 4.2. TTX and Analogues Extraction and Analysis

#### 4.2.1. Materials

The LC–MS grade solvents (water, acetonitrile, and methanol) were purchased from Carlo Erba (Milan, Italy). The LC–MS grade acetic acid, formic acid, ammonia hydroxide (25%), and ammonium formate; as well as the ENVI-Carb SPE cartridges (250 mg/3 mL volume) were purchased from Sigma-Aldrich (Darmstadt, Germany).

A certified reference standard (CRM), containing certified concentrations of tetrodotoxin (TTX), 21.0 ± 1.3 µg/g; 4,9-anhydroTTX, 5.44 ± 0.40 µg/g; and 4-*epi*TTX, 1.67 ± 0.15 µg/g; in aqueous acetic acid (1 mM), pH 3.91, was purchased from CIFGA Laboratorio S.A. (Lugo, Spain).

#### 4.2.2. Sample Extraction

The samples were homogenized with an Ultra-Turrax (T 25 easy clean digital, IKA-Werke GmbH & Co. KG, Staufen im Breisgau, Germany). Sample extraction was carried out by following the Standard Operating Procedure of the European Union Reference Laboratory for Marine Biotoxins for the determination of TTX [30,56]. In summary, samples were homogenized with 1% acetic acid, vortexed for 3 min, boiled in water for 5 min, cooled to room temperature, vortexed for another 3 min, and centrifuged for 10 min at 2200 g and 15 °C (Mega Star 600 R, VWR, Avantor, Radnor Township, PA, USA). Following centrifugation, ammonium hydroxide (0.025% *v*/*v*) was added to the supernatant, which was then cleaned using solid-phase-extraction (SPE). The SPE procedure was performed manually as follows: the SPE cartridges were conditioned with 3 mL acetonitrile/water/acetic acid (20:80:1 *v*/*v*/*v*), followed by 3 mL of water/ammonium hydroxide solution 25% (1000:1 *v*/*v*), with both solutions eluting to waste; then, 500 µL of sample extracts were loaded onto the conditioned cartridges, washed with 700 µL of Milli-Q water, which were also both eluted to waste; finally, toxins were eluted into Eppendorf tubes with 2 mL acetonitrile/water/acetic acid (20:80:1 *v*/*v*/*v*). The eluates were diluted (dilution factor of 4) with acetonitrile before analysis.

#### 4.2.3. LC–HRMS Conditions

The samples were analyzed by liquid chromatography–high-resolution mass spectrometry (LC–HRMS). The analysis was carried out using an Ultimate 3000 UHPLC system coupled to an Orbitrap Elite mass spectrometer (Thermo Fisher Scientific Inc., Waltham, MA, USA) equipped with a heated electrospray ionization source (HESI-II). The TTX and analogues were separated using an ACQUITY Premier BEH Amide (2.1 × 100 mm, 1.7 μm, Waters, Milford, MA, USA) at 35 °C. Samples were held in the autosampler at 4 °C. The mobile phase was composed of water with 0.1% formic acid and 10 mM ammonium formate (A) and acetonitrile with 0.1% formic acid and 2% 100 mM ammonium formate solution (B). The gradient (in *v*/*v*%) started with 5% of B and increased linearly to 95% in 11 min. This composition was maintained for 1 min and then returned to 5% of B in 1 min and maintained at this composition for 2 min before the next run [30]. The flow rate was 0.3 mL/min, and the injection volume was 10 µL. Data were acquired under positive (ESI+) polarity using the following ionization parameters: spray voltage, 3.8 kV; sheath gas, 40 arbitrary units; auxiliary gas, 10 arbitrary units; heater temperature, 300 °C; capillary temperature, 325 °C; and S-Lenses RF level, 69.06%. The LC–HRMS acquisition was performed under full-scan with the *m*/*z* ranging between 100 and 500. The (HCD) spectra of TTX analogues were obtained by running the system under product ion scan by fragmentation of the ion of each analogue over the entire chromatographic separation (LC–HRMS^2^) and detection from 50 to 350 *m*/*z*, using a normalized collision energy (CE) of 45 for deoxyTTX and 70 for the unknown TTX epimer and trideoxyTTX.

#### 4.2.4. Quantitation

The LC–HRMS quantitation was performed by generating accurate mass-extracted ion chromatograms (AM-XIC) obtained from the full-scan positive (ESI+) profiles using the exact mass (5 decimals) of each TTX analogue protonated molecular ion [M+H]^+^ (see Table 1), and a mass extraction window of ±5 ppm. The assignments of the TTX analogues were based on the exact masses, retention times and the fragmentation spectra obtained by HCD (Figure 2). The obtained concentrations of TTX analogues were estimated from the MS response to TTX standard. All other general MS parameters were adjusted to ensure an optimum signal for the TTX standard.

Quantification was performed by preparing matrix-matched calibration curves, with five concentration points, in blank crab extract. A working solution containing approximately 2 µM of TTX, 0.55 µM of 4,9-anhydroTTX, and 0.16 µM of 4-*epi*TTX was prepared. The tissue matrices were spiked with 2, 5, 10, 20, and 40 µL of the working solution per 200 µL of the matrix. For the determination of the concentration in µg TTX equivalent (eq)/kg, the relative potencies of 0.16 and 0.01 were used for the unknown TTX epimer and deoxyTTX/trideoxyTTX, respectively; as recommended by the EFSA for 4-*epi*TTX and 5,6,11-deoxyTTX/5,6,11-trideoxy TTX [3,31,32,33].

The limits of detection (LOD) and quantification (LOQ) were calculated from the standard deviations (SD) obtained after five injections of each blank matrix spiked with the second-lowest concentration (3 × SD and 10 × SD, respectively). The matrix effect (ME) was obtained after three injections of the third-lowest concentration standard solution and each non-contaminated blank matrix spiked with this concentration and calculated using the equation $ME\;\left(\%\right)=\frac{B}{A}\times 100$, where A is the average peak area of the standard solution and B represents the average peak area in the extract spiked with the same concentration.

## Figures and Tables

**Table 1:** Structure of protonated tetrodotoxin and its analogues and correspondent exact masses.

Structure	Analogue	R1	R2	R3	R4	Molecular Formula	[M+H]^+^ Exact Mass
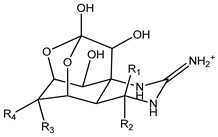	TTX	H	OH	OH	CH_2_OH	C_11_H_17_N_3_O_8_	320.10884
	4-*epi*TTX	OH	H	OH	CH_2_OH	C_11_H_17_N_3_O_8_	320.10884
	11-deoxyTTX	H	OH	OH	CH_3_	C_11_H_17_N_3_O_7_	304.11393
	11-norTTX-6(R/S)-ol	H	OH	H	OH	C_10_H_15_N_3_O_7_	290.09828
	6,11-dideoxyTTX	H	OH	H	CH_3_	C_11_H_17_N_3_O_6_	288.11901
	11-oxoTTX	H	OH	OH	CHO	C_11_H_17_N_3_O_9_	336.10376
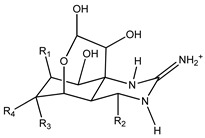	5-deoxyTTX	H	OH	OH	CH_2_OH	C_11_H_17_N_3_O_7_	304.11393
	5,11-dideoxyTTX	H	OH	H	CH_3_	C_11_H_17_N_3_O_6_	288.11901
	5,6,11-trideoxyTTX	H	OH	H	CH_3_	C_11_H_17_N_3_O_5_	272.12410
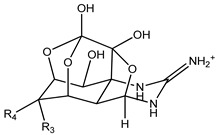	4,9-anhydroTTX	─	─	OH	CH_2_OH	C_11_H_15_N_3_O_7_	302.09828
	6-*epi*-4,9-anhydroTTX	─	─	CH_2_OH	OH	C_11_H_15_N_3_O_7_	302.09828

## Data Availability

The data generated in this study are contained within this article and its Supplementary Materials.

## Supplementary Materials

The following supporting information can be downloaded at: https://www.mdpi.com/article/10.3390/md21060320/s1, Figure S1: Accurate mass-extracted ion chromatograms (AM-XIC) generated from the LC-HRMS full-scan positive mode (ESI+) chromatograms using the theoretical TTX m/z 320.10884 with a ± 5 ppm extraction window of TTX certified reference standard (CRM) in acetonitrile/water/acetic acid solution, containing 4-*epi*TTX and TTX (A), a real crab sample, soft tissues of European fiddler crab harvest near Faro beach, containing an unknown TTX epimer (B), and the same real sample skipped with the TTX CRM, containing an unknown TTX epimer and TTX (C).
