# Supplementary material for: New Vectors of TTX Analogues in the North Atlantic Coast: The Edible Crabs Afruca tangeri and Carcinus maenas
Source: Mar Drugs. 2023 May 25;21(6):320. doi: 10.3390/md21060320 (PMC10305652; doi:10.3390/md21060320)
Supplement: Supplementary file 1 [file marinedrugs-21-00320-s001.zip › marinedrugs-2378432-supplementary.pdf]

Supplementary Materials

New vectors of TTX analogues in the North Atlantic Coast: the edible crabs *Afruca tangeri* and *Carcinus maenas*

Sandra Lage [1,*], Felicitas ten Brink [1,2], Adelino V. M. Canário [1] and José P. Da Silva [1]

[1] Centre of Marine Sciences (CCMAR/CIMAR LA), University of Algarve, Campus de Gambelas, 8005-139, Faro, Portugal
[2] Energy and Environment Institute, School of Environmental Sciences, University of Hull, Hull, HU6 7RX, United Kingdom
* Correspondence: smlage@ualg.pt

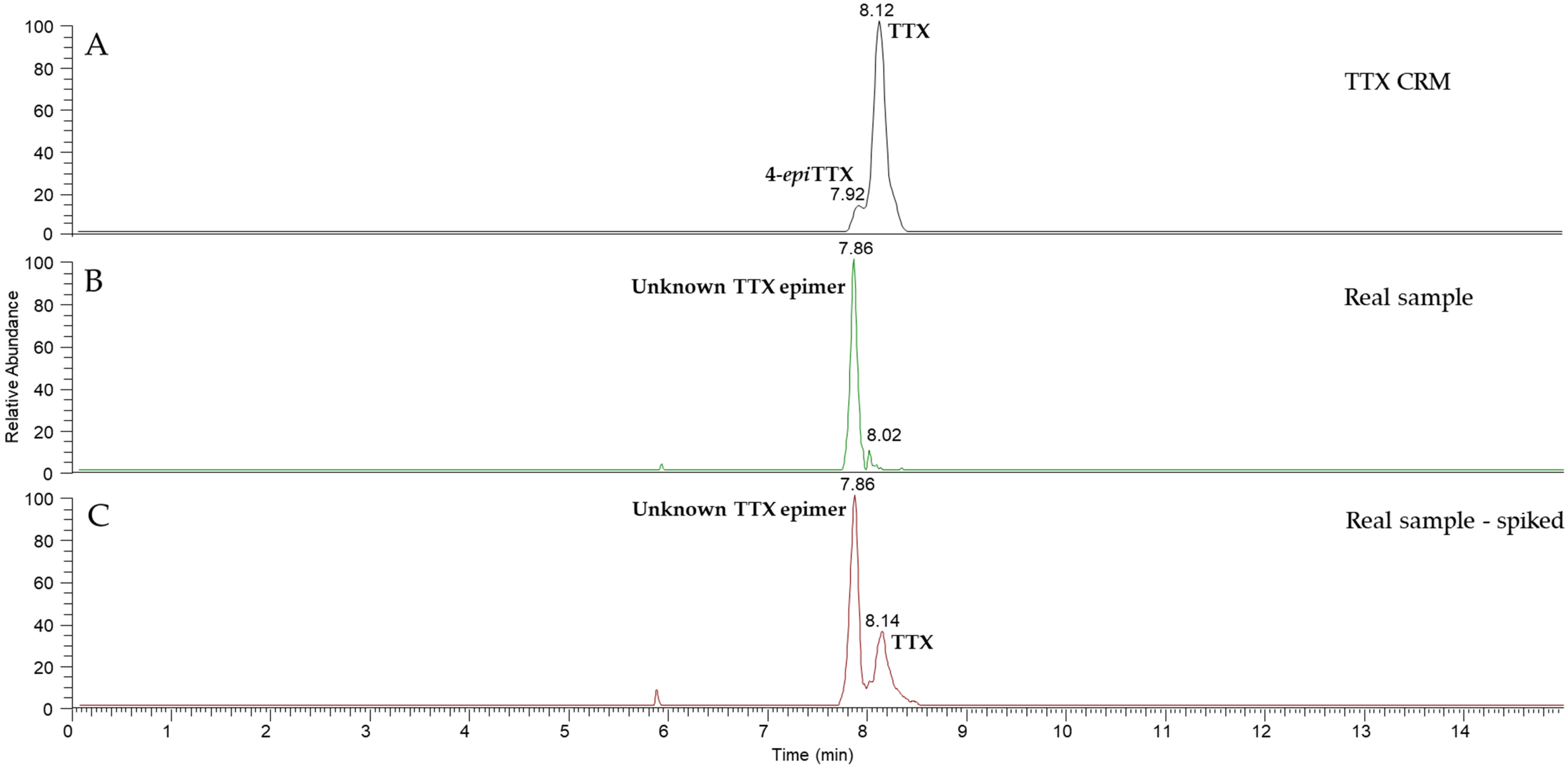

Figure S1. Accurate mass-extracted ion chromatograms (AM-XIC) generated from the LC-HRMS full-scan positive mode (ESI +) chromatograms using the theoretical TTX *m/z* 320.10884 with a ± 5 ppm extraction window of TTX certified reference standard (CRM) in acetonitrile/water/acetic acid solution, containing 4-*epi*TTX and TTX (**A**), a real crab sample, soft tissues of European fiddler crab harvest near Faro beach, containing an unknown TTX epimer (**B**), and the same real sample skipped with the TTX CRM, containing an unknown TTX epimer and TTX (**C**).
